# Supplementary material for: A role for CBFβ in maintaining the metastatic phenotype of breast cancer cells
Source: Oncogene. 2020 Jan 31;39(12):2624–37. doi: 10.1038/s41388-020-1170-2 (PMC7082223; doi:10.1038/s41388-020-1170-2)
Supplement: Supplementary file 1 — Supplementary Figure Legends [file 41388_2020_1170_MOESM1_ESM.docx]

**Figure S1**

**Generation of stable MDA-CBFβ-ER cell lines and MDA- pcDNA3.1/H cell line.** (A) Western blot showing CBFβ-ER and CBFβ expression. The total cell lysates were derived from wild type MDA-MB-231 cells and six clones of MDA-CBFβ-ER cells followed by western blots against an anti-CBFβ antibody. The upper panel showed the CBFβ-ER and the middle panel presented endogenous CBFβ. Clone numbers 2, 4 and 6 all had the expression of CBFβ-ER fusion protein and only the wild-type cells showed the endogenous CBFβ protein. Clone 6 was selected for subsequent experiments. (B) Expression of CBFβ-ER was analyzed by western blots using CBFβ antibody in total cell lysates from seven clones of control cell line MDA-pcDNA3.1/H. There was no CBFβ-ER band in the control cell lines. Clone number 1 was selected for further analysis. β-Tubulin was used as a loading control.

**Figure S2**

Depletion of CBFβ or RUNX1 in MDA-MB-468 inhibits invasion. Cells were transfected with siCBFβ siRUNX1 or siNS. 48 hours after transfection, mRNA levels of CBFβ and RUNX1 were quantified (A) and protein (B) to confirm knockdown. (C) Knockdown of CBFβ or RUNX1 leads to a decrease in invasion. (D) qRT-PCR analysis was performed on EMT marker genes 72 hours after transfection. Data is presented as mean ± SEM from three independent experiments performed in triplicates. NS (p>0.05) indicates none significant differences, *(p<0.05) and *** (p<0.001) indicate significant differences compared to the non-specific control analysed by the Student’s t-test.

**Figure S3**

**Additional controls for tamoxifen inducible system.** Induction of CBFβ-ER with 4OH-T restores the mesenchymal phenotype in 3D culture but no effect is seen in pcDNA3.1/H control cells. The GFP expressing cells were visualised by fluorescence microscopy and DAPI staining. Scale bars are 200μm.

**Figure S4**

**RUNX1 knockout leads to a loss of the mesenchymal phenotype in MDA-MB-231 cells.** (A) A double nickase CRISPR-Cas9 system was used to knockout the start of the RUNX1 gene in MDA-MB-231 cells^23^. Two clones with complete RUNX1 loss were generated and a western blot shows the levels of RUNX1, RUNX2 and CBFβ in these cells with β-tubulin used as a loading control. (B) RUNX1 knockout leads to a loss of the mesenchymal phenotype of MDA-MB-231 cells. WT-MDA-MB-231 and RUNX1^-/-^ cells were grown in a 3D Matrigel. Cells were fixed after 14 days and nuclei stained with DAPI (blue) and phalloidin (red). The parental cells showed the stellate mesenchymal pattern after 14 day. RUNX1^-/-^ cells formed discrete clusters. A decrease in invasive capacity of RUNX1 knockout cells was determined using a Boyden chamber Matrigel assay. Scale bars are 50μm (C) A decrease in invasive capacity of RUNX1 knockout cells was determined using a Boyden chamber Matrigel assay. (D) Mammosphere forming efficiency was decreased in RUNX1 knockout cells. Mammospheres were counted after 5 days growth in non-adherent culture. (E) Loss of RUNX1 causes a reduction in mesenchymal markers and increased expression of epithelial markers. RNA from parental MDA-MB-231 and RUNX1^-/-^ cells were subject to qRT-PCR. Acidic ribosomal phosphoprotein P0 (RPLO) mRNA was used for normalisation and relative values of each marker gene mRNA levels are shown.

**Figure S5**

**RUNX2 depletion leads to a loss of the mesenchymal phenotype in MDA-MB-231 cells.** A) Western blot showing levels of RUNX2, RUNX1 and CBFβ in RUNX2 depleted MDA-MB-231 cells. HeLa cells used as a Runx1/Runx2 low cell line control. Lamin B1 was used as a loading control. (B) WT-MDA-MB-231 and MDA-shRUNX2 cells were grown in 3D Matrigel. Cells were fixed after 14 days and nuclei stained with DAPI (blue). GFP (green) was stably expressed in all cell lines. The parental cells showed the stellate mesenchymal pattern after 14 day. MDA-shRUNX2 cells formed discrete clusters. (C) Scratch assays showing inhibition of migration in MDA-shRUNX2. Live images were taken every 20 mins for 48 hours.

(D) Invasive capacity was reduced in shRUNX2 cells. Matrigel invasion assay showing the invasion rates following loss of RUNX2 after 24 hours culture. (E) Mammosphere forming efficiency was also reduced in shRUNX2 cells compared to control cells. Mammospheres were counted after 5 days growth in non-adherent culture. (F) Loss of RUNX2 causes a reduction in mesenchymal markers and increased expression of epithelial markers. qRT-PCR was performed for epithelial and mesenchymal markers in MDA-shNS or MDA-shRUNX2. Acidic ribosomal phosphoprotein P0 (RPLO) mRNA was used for normalisation and relative values of each marker gene mRNA levels are shown.

**Figure S6**

**Generation CBFβ-negative MDA-MB231 using CRISPR**. (A) A double nickase CRISPR-Cas9 system was used to knockout the start of the CBFβ gene in MDA-MB-231 cells. One clone was generated with complete CBFβ loss and CBFβ-Flag was then re-expressed in this as a rescue. A western blot shows the levels CBFβ in these cells with β-tubulin used as a loading control. (B) CBFβ loss leads to a loss of the mesenchymal phenotype of these cells, which can be rescued *via* CBFβ-Flag re-expression. Cells were grown for 14 days and then imaged using a brightfield microscope. Scale bars are 100μm (C) A decrease in invasive capacity of CBFβ^-/-^ cells was determined using a Boyden chamber Matrigel assay. This can be rescued via CBFβ-Flag re-expression. (D) Mammosphere forming efficiency was also decreased in CBFβ^-/-^ cells and was rescued with re-expression of CBFβ-FLAG. Mammospheres were counted after 5 days growth in non-adherent culture. (E) Loss of CBFβ causes a reduction in mesenchymal markers. RNA from MDA-MB-231 control and CBFβ^-/-^ cells were subject to qRT-PCR. Acidic ribosomal phosphoprotein P0 (RPLO) mRNA was used for normalisation and relative values of each marker gene mRNA levels are shown.
