# Supplementary figures and images for: A role for CBFβ in maintaining the metastatic phenotype of breast cancer cells
Source: Oncogene. 2020 Jan 31;39(12):2624–37. doi: 10.1038/s41388-020-1170-2 (PMC7082223; doi:10.1038/s41388-020-1170-2)

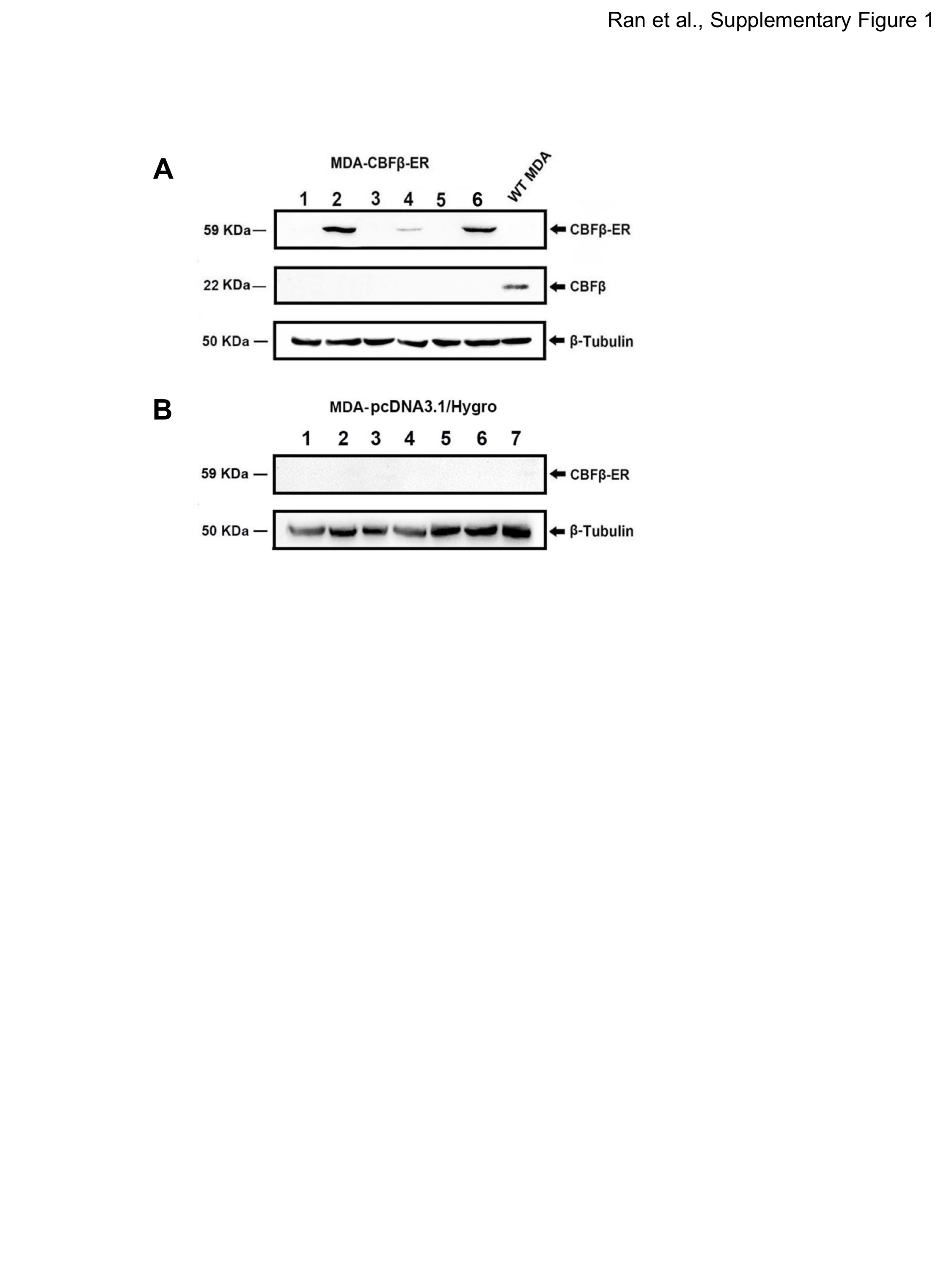

Supplement: Supplementary file 2 — Supplementary Figure 1 [file 41388_2020_1170_MOESM2_ESM.tif]

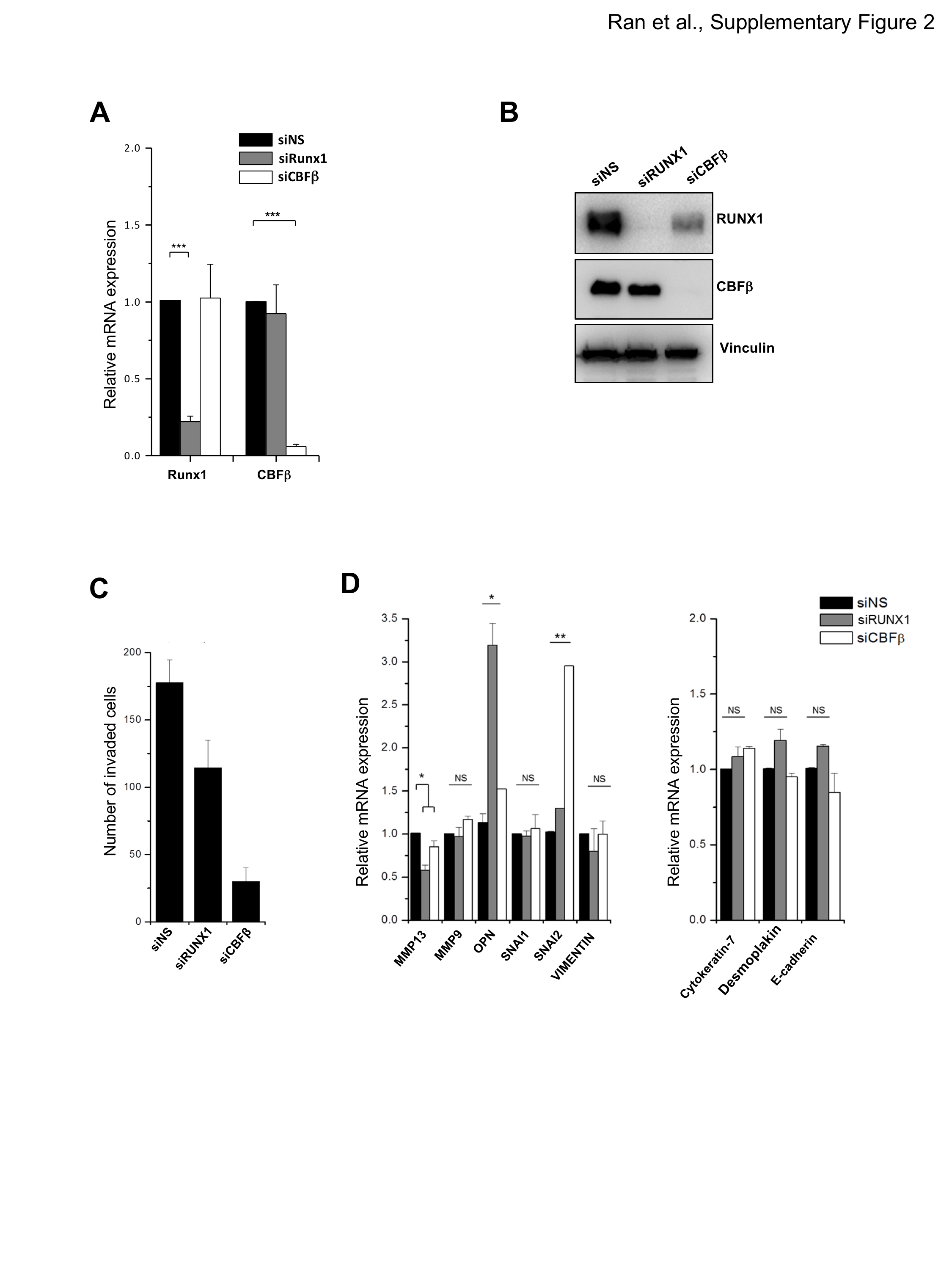

Supplement: Supplementary file 3 — Supplementary Figure 2 [file 41388_2020_1170_MOESM3_ESM.tif]

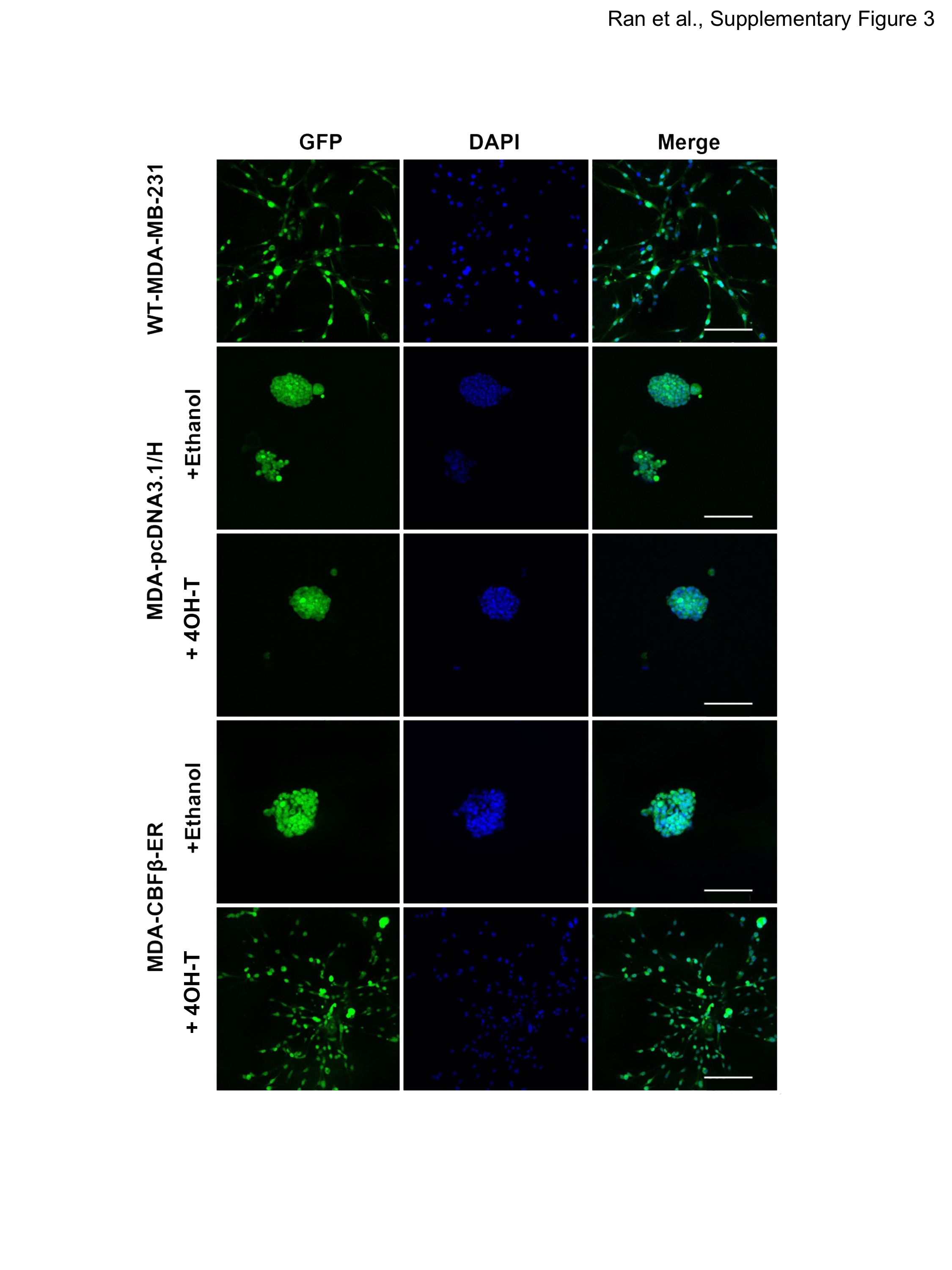

Supplement: Supplementary file 4 — Supplementary Figure 3 [file 41388_2020_1170_MOESM4_ESM.tif]

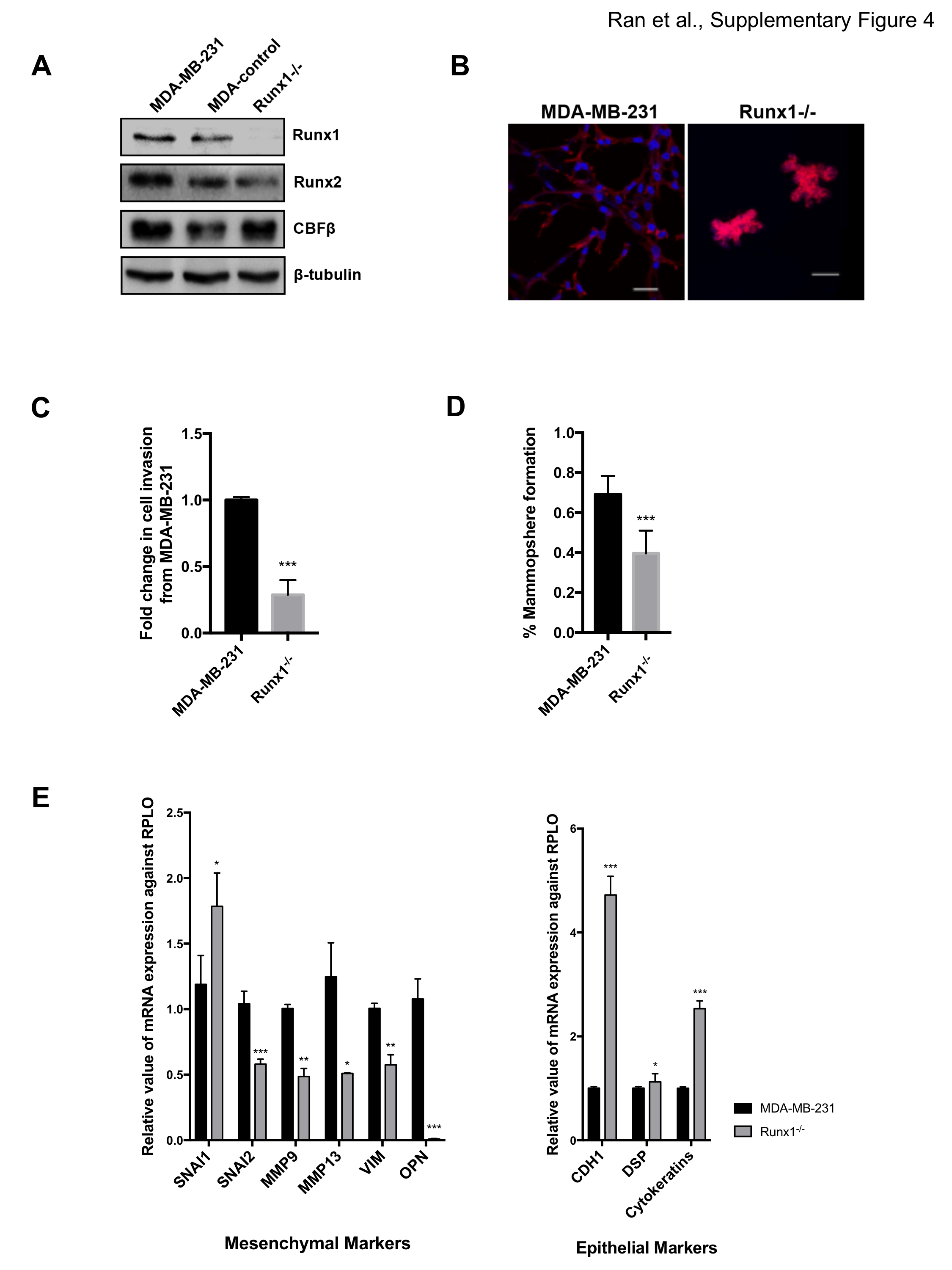

Supplement: Supplementary file 5 — Supplementary Figure 4 [file 41388_2020_1170_MOESM5_ESM.tif]

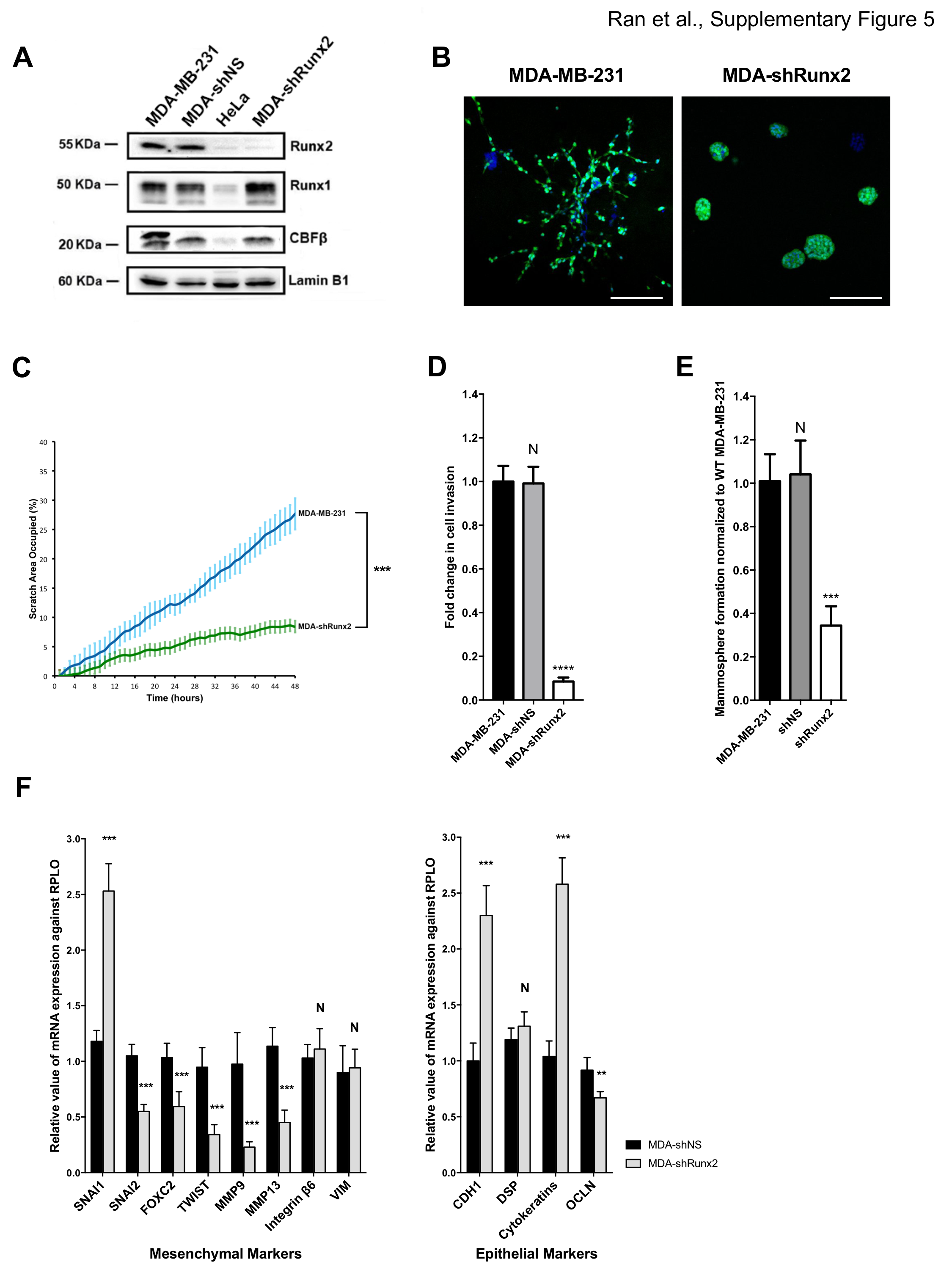

Supplement: Supplementary file 6 — Supplementary Figure 5 [file 41388_2020_1170_MOESM6_ESM.tif]

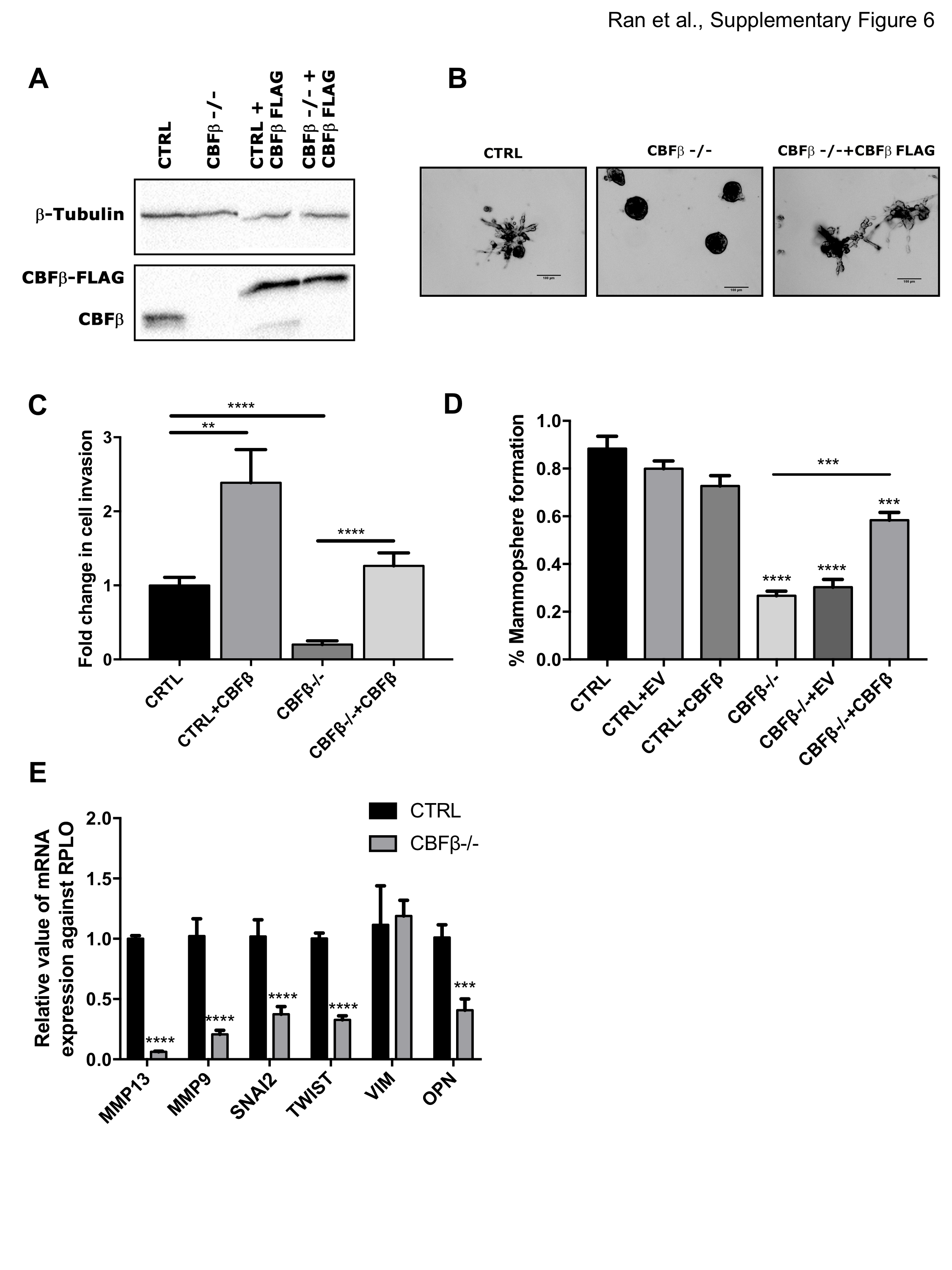

Supplement: Supplementary file 7 — Supplementary Figure 6 [file 41388_2020_1170_MOESM7_ESM.tif]

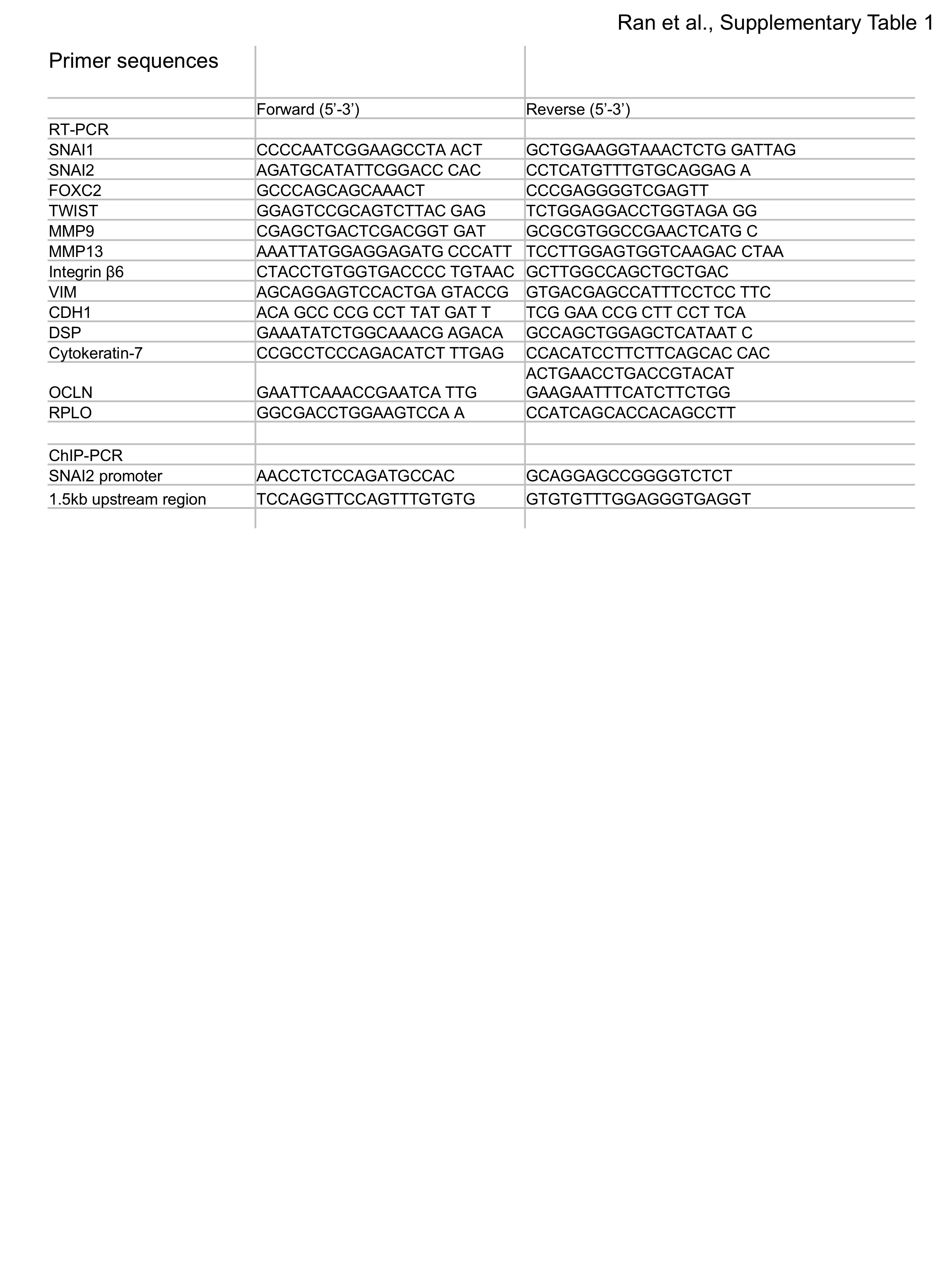

Supplement: Supplementary file 8 — Supplementary Table 1 [file 41388_2020_1170_MOESM8_ESM.tif]
